# Needs, Experiences, and Hopes for Aging Futures among Older Adults in the LGBTQ Communities: A Qualitative Study in Israel

**DOI:** 10.1007/s10508-024-02938-x

**Published:** 2024-07-15

**Authors:** Daniel Sperling

**Affiliations:** The Cheryl Spencer Department of Nursing, University of Haifa, 199 Aba Khoushy Ave., Mount Carmel, 3498838 Haifa, Israel

**Keywords:** LGBTQ, Aging, Israel, Stigma, Loneliness, Sexual orientation

## Abstract

Older lesbian, gay, bisexual, trans, and queer (LGBTQ) individuals tend to live alone, mostly without children and with scarce support from nuclear family members or biological kin. Moreover, traditional resources may not suit their specific end-of-life care needs. While studies have examined these topics in general, they lack focus on end-of-life needs, care, and planning in Israel. Moreover, research on this topic among members of LGBTQ communities is specifically lacking. This study, therefore, aimed at identifying and understanding the attitudes, perceptions, and meanings of older LGBTQ individuals in Israel regarding their needs and challenges, as they age and near end of life. The phenomenological qualitative research methodology was applied, following the interpretive approach. Twenty-one middle-aged and older LGBTQ individuals in Israel, aged ≥ 55, participated in the study. In-depth semi-structured interviews, conducted from November 2020 to April 2021, were audio-recorded, transcribed, and de-identified. Five themes emerged from the interviews: (1) Experiences of loneliness, marginalization, and trauma, and coping through liberation; (2) ageism and exclusion of older adults; (3) elastic and challenging relationships; (4) end of life as reverting into the closet and heteronormativity; and (5) death as a source of generativity and creativity. The study demonstrates that loneliness is an existential experience, exacerbated by the intersectionality of LGBTQ communities. In turn, chosen family members play a minimal role in the end-of-life care of their loved ones. While conveying ambivalence toward social services and housing for the aging, participants in this study expressed fear of being discriminated against and having to re-enter the closet as they age. Ageism and end of life do not represent finality and extinction, yet instead, signify hope and revival. Following Sandberg and Marshall’s (2017) concept of queering aging futures, this study refines our understanding of life courses, demonstrating that living and thriving in old age could be positive and desirable. As such, ageism and end of life do not necessarily represent finality and extinction, and may instead signify hope and revival. The unique challenges associated with family and social support of older adults who are LGBTQ members, and their implications on care, deserve further research and are important for practice.

## Introduction

Older members of the lesbian, gay, bisexual, trans, and queer (LGBTQ) communities are twice as likely to live alone than others, most probably without children and with scarce support by nuclear family or biological kin (Institute of Medicine, 2011). They may also have a greater need for care, with fewer traditional resources for end-of-life care provision (Cottrell, 2020; de Vries & Gutman, 2016; de Vries et al., 2019). Indeed, these populations tend to report higher rates of medical problems, disabilities, emotional burden, and financial instability compared to heterosexual and gender-conforming people of the same age group (Bailey et al., 2022; de Vries & Gutman, 2016; Flatt et al., 2022). They may also suffer from oppression, marginalization, and violence (Flatt et al., 2022)—as well as from stigmas, homophobia, and ageism—from within and outside their communities (Orel, 2014). The term *ageism* refers to stereotypes, prejudice, or discrimination against (or in favor of) individuals, based solely on their chronological or perceived older age (Iversen et al., 2009)—from both within and outside their communities (Orel, 2014). It is important to also address the concept of internalized homophobic/gay ageism. This minority stress construct differs from perceived ageism and from internalized homophobia; instead, it represents the individual’s feelings of denigration or depreciation, following their aging as a person from a sexual or gender minority (Wight et al., 2015). Such feelings often increase the risk of anxiety, depression, somatization, and even suicidal ideation (Pereira, 2021; Pereira et al., 2020; Salway & Gesink, 2018); these negative emotions are also known to decrease well-being, resulting in decreased subjective experience of happiness and life satisfaction, psychological functioning, and self-realization (Semlyen et al., 2016).

In recent years, positive changes have been seen in social attitudes toward sexual and gender minorities (SGMs), (OECD, 2020). This term refers to populations of lesbian, gay, bisexual, and transgender individuals, and to people whose sexual orientation, gender identity and expressions, or reproductive development varies from traditional, societal, cultural, or physiological norms (National Institute on Minority Health & Health Disparities, 2016; Nikzad et al., 2024; Suen et al., 2020). However, despite such desirable changes, these populations may still lack social support within the LGBTQ communities themselves, rendering them dealing with social exclusion and loneliness. This reality is especially prevalent among those with less education, friends and nuclear family support, and connectedness to the LGBTQ communities (Ribeiro-Gonçalves et al., 2022). As a result, adult members of the LGBTQ communities face unique end-of-life barriers that may perpetuate a heteronormative culture; these may also negatively impact the care that they receive as they age, including a culture of “don’t ask, don’t tell.” As a result, such individuals may incur unmet healthcare needs, marriage and gender-recognition laws (and other forms of discriminative legislation), institutional policies regarding expenses and insurance in end-of-life care, and even inapplicable bereavement support services (Lintott et al., 2022).

Studies show that poor health conditions, lack of community support, and social isolation may significantly impair quality of life for LGBTQ members, hindering their successful aging and ability to deal with end-of-life challenges (de Vries et al., 2019; Flatt et al., 2022; Ribeiro-Gonçalves et al., 2022). The term refers to the high physical, psychological, and social functioning of older people (Bowling & Dieppe, 2005; Rowe & Kahn, 1987). Combined with financial instability, older LGBTQ individuals may face dramatic difficulties regarding end-of-life decisions, advance medical directives, and care planning (de Vries & Gutman, 2016; Hughes & Cartwright, 2014). Additional obstacles may include inadequate care and biological family support (Roos Pijpers & Sandra Van Beek, 2021), insufficient knowledge of end-of-life legal rights, and advance care planning, as well as difficulty appointing a decision maker or guardian (Cartwright et al., 2012).

Studies also explore that older LGBTQ members may strive to leave personal legacies that will impact how they will be remembered, as well as broader legacies pertaining to improved LGBTQ rights. They often seek recognition from younger generations regarding their contribution to the safety and improved rights of the members of the community (Lee et al., 2017). As they age, they also seek an inclusive and safe environment, where they can continue to feel connected to a community (Putney et al., 2018). A review of related studies indicates that older LGBTQ members are concerned about long-term services and support, and about having someone to advocate for them at their end of life (Caceres et al., 2020). They may also be worried about being discriminated against, due to their sexual orientation or gender identity while being cared for by healthcare professionals, and especially in relation to moving into sheltered accommodation with their same-sex partner (Mahieu et al., 2019).

Older trans people have additional concerns, such as their gender identity being acknowledged, receiving intimate care and being dependent on others, and even issues of abuse—especially in long-term care (Valenti et al., 2020). A large-scale study did not find sexual orientation to play a significant role in the shaping of preferences regarding moving into a nursing home (Buczak-Stec et al., 2021). Yet LGBTQ members in long-term care settings fear that their sexual identity will become invisible or ignored, and even articulate the need for increased staff training (Burton et al., 2023; Fasullo et al., 2021).

In Israel, a Jewish and democratic state, members of LGBTQ communities may encounter numerous obstacles that prevent them from comprehensively and equally participating in society—even in comparison with members of similar communities in other democratic states (Ilany & Ilany, 2021). Yet these individuals often find ways to become a part of the national collective—especially through the mandatory 2–3-year military service, as well as the country’s exceptional pronatalist policy and social attitudes toward procreation (Hartal & Sasson-Levy, 2023). Older LGBTQ individuals often lack stable family support, turning instead to close friends, who become their unofficial family. They report twice as much loneliness as the general population and suffer from depression four times greater than their same-aged peers (Shnoor & Berg-Warman, 2019). Relationships between older LGBTQ individuals are often concealed, until one of the partners becomes the other’s main caregiver—rendering them concerned about social reactions to their non-heteronormative care relationship (Alon & Yaacov, 2012). Despite significant developments in LGBTQ rights and the emergence of a normative LGBTQ culture in Israel in recent years (Hartal, 2020), represented also in all modern civil rights system (except of marriage and full family rights) (Pizmony-Levy et al., 2019), older LGBTQ adults still report conservative negative attitudes toward them.

Unexpectedly, they even experience disrespect, humiliation, and rejection by younger LGBTQ individuals (Waldmann et al., 2011). As such, older members often live in a constant state of conflict and tension between oppression (reflected through ageism and homophobia) and liberation (reflected through open expressions of sexual orientation and lack of depression and loneliness) (Meri-Esh & Doron, 2009).

While studies have examined end-of-life needs, care, and planning in Israel among older populations in general (e.g., Meri-Esh & Doron, 2009; Shnoor & Berg-Warman, 2019), the literature lacks such research specifically in relation to members of LGBTQ communities. As such, this study strives to identify and understand the attitudes, perceptions, and meanings of older individuals from LGBTQ communities in Israel regarding the challenges that they face as they age, as well as their end-of-life needs, especially in terms of their receiving quality care and support (Ben Natan et al., 2010). The study particularly places an emphasis on these individuals’ choices, obstacles, and uncertainties. To understand the participants’ risk and protective factors regarding their wellbeing and current needs, especially as they age and even near death, the study follows the Health Equity Promotion Model and Iridescent Life Course approach (Fredriksen-Goldsen et al., 2019, 2023). The model helps understand and contextualize the experiences and perceptions of older adults in these communities and their meanings as being shaped, interacted, and negotiated at all levels (personal, interpersonal, community-based, and institutional) by and as a result of cataclysmic historical events. Examples of such events include the elimination of public discourse on homosexuality, social and medical targeting of these individuals and eventually making their identities public and acceptable through discourse of pride and liberation and an appeal to civil rights.

## Method

### Participants

The 21 individuals who participated in this study were aged 55 or older, members of the LGBTQ communities in Israel, and defined themselves as lesbian, gay, bisexual, trans, and/or queer. The sample size is considered acceptable for the chosen research design, as it is determined by the context and information that is provided by the sample. It is supported by the concept of information power whereby the interviews were assessed—each interview separately and as a whole—by the following criteria: study aim, sample specificity, use of established theory, quality of dialogue, and analysis strategy (Malterud et al., 2016).

### Measures and Procedure

The research followed the Interpretive Phenomenological Approach (IPA) (Lopez & Willis, 2004). IPA focuses on the participants' claims, explanatory logic, biographical contexts, and cultural frameworks of knowledge. It aims at understanding what the participants convey, following their experiences and the meanings that they attach to such events. This research design was chosen as it enables in-depth understandings and insights into the participants’ tacit knowledge, shared meanings, and informal norms—regarding aging in general and among SGMs in particular. In line with queer theory (Butler, 2011; Hurd et al., 2022; Seidman, 1996), the current study assumes that the identities of individuals from LGBTQ communities develop and are reconstructed through nonlinear processes of socialization. They also emerge and evolve following negotiations of social constructs over the course of their lives—including as they age or near their end of life. Applying queer theory to the current study enables reference to and discussion of a range of so-called binary terms—such as heterosexuality–homosexuality, constraint–freedom, and commitment–boundless relationships—while transitioning within and between these terms in a recurring and flexible process. This is reflected in the negotiation and self-negotiation of the understanding and meaning of these terms—between and within the researcher and the participants.

The study took place in the participants’ preferred location, such in the privacy of their home or at a café (2 participants) and via the Zoom platform (19 participants)—on a preset date and time of their convenience. To protect the participants' in the utmost manner, no other individuals were present during the interviews.

The participants were recruited through convenience and snowball sampling, and through requests to participate via relevant Facebook groups and support groups for older LGBTQ adults in four major cities in Israel (Tel Aviv, Jerusalem, Haifa, and Beer-Sheva). Participant recruitment was conducted until thematic saturation was achieved. The point of saturation is reached when the data are adequately rich in quality and in quantity. This is when there is sufficient information for replicating the study—should additional data be obtained, and when further coding is no longer feasible (Fusch & Ness, 2015). Initially, the researcher encountered certain challenges in recruiting older transgender participants for this study; these individuals tended to perceive the researcher’s phone call as suspicious, potentially malicious even. Yet following preliminary conversations, where more detailed explanations about the research were provided, they then agreed to participate in the study. The actual interviews were conducted at a later date, so as not to be impacted by the participants’ initial suspicions Moreover, only one older bisexual person took part in the study. The possible under-representation of older bisexual individuals in this study may result from a range of reasons. For example, they may be less likely to identify themselves as such, compared to other LGBTQ sub-groups in Israel; alternatively, people who identify as bisexual may be less ingrained in the LGBTQ communities in Israel. In turn, older people who define themselves as bisexual may not have been as exposed to the request to participate in this study as others.

Twenty-one in-depth, semi-structured interviews were conducted with older LGBTQ members between November 2020 and April 2021. The interviews were conducted by the researcher in Hebrew and lasted 70–145 min. The participants were assured complete anonymity (pseudo-initials are used throughout the article); with the participants’ informed consent, each interview was recorded via the Zoom platform or a tape recorder, transcribed by a professional transcriber, and de-identified.

An interview guide was developed for this study, based on the relevant literature. The interviews included questions relating to the participants’ demographic characteristics; sexual orientation and participants' social and organizational surroundings; and perceptions of aging and end of life (see Appendix).

### Data Analysis

The data were analyzed through a multi-level and cohesive process, conducted in the following stages: (1) bracketing away from the participants’ lived experiences; (2) reading and annotating the major issues and questions in the transcripts; (3) preparing a list of codes that are closely derived from the participants’ words and wording; (4) grouping these codes into concepts, using more extensive interpretation, and then into themes; (5) writing textural descriptions of what the participants experienced in relation to each phenomenon, concept, or theme, including verbatim examples; (6) creating structural descriptions by reflecting on the setting and context in which the participants experienced the phenomenon; and (7) writing a composite description of the researched phenomenon based on the textural and structural descriptions (Creswell & Poth, 2016).

The findings were arranged in final categories and themes using the Atlas.ti 9 Program. To increase trustworthiness of the analysis, the data were independently analyzed by the author and by a research assistant. This was followed by reflexivity and shared discussions of the major findings, including drawing links and connections in cases of differing interpretations.

## Results

The participants’ main characteristics are presented in Table 1. Following the data analysis, the following five main themes emerged from the interviews: (1) Experiences of loneliness, marginalization, and trauma, and coping through liberation; (2) ageism and exclusion of older adults; (3) elastic and challenging relationships; (4) end of life as reverting into the closet and heteronormativity; and (5) death as a source of generativity and creativity. The order of the themes as presented in the following sections is derived from the need to provide a coherent narrative and is in line with the biographical / life course narrative analysis approach.

**Table 1:** Participants’ demographics

Gender (as stated)	9 Women (3 transwomen); 11 men; 1 queer
Age (years)	67 (average); 55–83 (range)
Sexual orientation	10 Gays; 8 Lesbians; 1 Bisexual; 2 others
Marital status	12 in an LGBTQ partnership (one married); 9 single; 7 divorced; 1 separated; 1 widow
Parental status	10 had biological children; 1 had a non-biological child; 1 had an adopted child; 9 had no children
Education	2 Ph.D.; 8 M.A.; 8 B.A.; 3 high-school education
Nationality	21 Jewish; 0 Arabs
Geographical area	9 Tel-Aviv and Central area; 7 Haifa and Northern area; 4 Jerusalem area; 1 Beer-Sheva and Southern area

### Theme 1: Experiences of Loneliness, Marginalization, and Trauma, and Coping Through Liberation

Many participants spoke of difficulties that they had encountered as they were learning about their personal sexual identity, while growing up in a very different era than today. The narratives specifically reflected an extended experience of loneliness, whose effect may still be felt. As Benny, a 61-year-old gay man, explained, “It was tough growing up as a gay man in the 1960s and 1970s. I married [a woman] but not because I wanted to. There was just no other alternative…there was no way to communicate with other [gays]. There were no phones.” Raphael, also a 60-year-old gay man, spoke along similar lines:

I started hanging out at the Independence Park [in Tel Aviv], because that was the only place to meet [other gay men]. There was no other place. There was just one bar, Bar 21...I went to that bar from the age of 17 wearing my youth-group uniform. I would always drag a youth-group leader with me because I was afraid to go alone.

These difficulties were especially enhanced for trans participants, as explained by Tzila, a trans woman:

When I was young, it was very difficult to be a trans woman…Everything [and everyone] was against me… starting with the police who arrested me. I was beaten up all the time... There were no psychologists, no committees, no doctors who cared for us…I took hormones like candies... and there was no one to supervise us… we were in the margins of society.

Some of the participants also mentioned earlier stages of their lives as having been traumatic and said they were still suffering from emotional and psychiatric pain, even finding it difficult to function—perhaps even more so later in life. Tzila, a trans woman, for example, conveyed such a reality:

I have a crying attack whenever I’m anxious about something. And then I speak to my other half about it, and they calm me down. It’s a kind of mechanism that I secretly developed when I was small. I had to because there was no one to do this for me…I’m afraid of people. People did bad things to me throughout my lifetime.

Jack, a 57-year-old bisexual man, revealed that he had sunk “into a kind of redness, an auto-depression…In fact, it has been difficult for me to function at any of my jobs over the past 10 years.”

Despite this heavy feeling and cost of past experiences, some participants expressed a strong sense of liberation, placing personal freedom on a pedestal, especially after coming out to oneself. Orit, a 62-year-old trans woman, used the metaphor of having been able to “leave the dangerous and frightening enclosed birdcage and fly out into the free world.” Similarly, Florine, a lesbian woman, explained:

That feeling of freedom, the feeling that I am doing what I want, that I decide for myself, as much as possible. It’s not complete, but it’s the day-to-day where I do what’s good for me. I wake up in the morning, I feel that I’m doing things that are meaningful to me. This is a big privilege. This freedom to do what you want. For me this is wonderful.

### Theme 2: Ageism and Exclusion of Older Adults

Throughout the interviews, this theme was most broadly discussed, as it was divided into the following four sub-themes: First, the findings reveal that old age is perceived as a relationship barrier, an object of distorted or inappropriate relationships within the LGBTQ communities. As explained by Koby, a 61-year-old gay man:

Today, being 60 years old, when you’re trying to meet people, is not easy…You can meet people when you’re 20-30, 35, but 61 is something…different types of people approach me…married people are sure that I have a home, a place to fuck...I’m old so it lowers their fear in general, fear of a relationship...Their sexual need is just a sexual need, that’s it. Then guys who are looking for money also approach me. Why? Because old people have to pay young guys who want money for sex. Or support…Then there are those who want to live with someone. A young person who wants to leave home and is looking for where to live. He knows that an old person will take him in. Finally, all sorts of complicated guys who think of me as a father figure, who seek guidance, they also approach me.

Second, old age is perceived as alien to sex in general and to sexual orientation in particular. Both Florine (72-year-old lesbian woman) and Moshe (75-year-old gay man) conveyed this notion:

You don’t see older gay or lesbian couples. They’re invisible. Why? Because when it comes to sex, if you’re talking about sexual preferences, it’s not something people think happens with old age. They don’t think there’s sex in old age. Sex is for the young (Florine)

There’s this addiction to sex in the gay community…there are gay people who enjoy their share of “fresh meat,” until one day they reach that age when there’s no more fresh meat, nobody wants them [anymore], and they find themselves all alone, and they have to deal with that. (Moshe)

The third sub-theme that emerged as part of this theme relates to old age as experienced through (sexual) communication within the LGBTQ communities, especially holding physical meaning. This was expressed by Vinny, a male activist in the gay community:

People don’t like the idea of dating within their own age group, which is ok, it’s legitimate. But…it’s a vicious cycle: when you’re young, you don’t want to mess about with older guys. When you’re old, you don’t want to mess around with older guys either. But then you’re insulted when someone 35 years younger than you doesn’t want to be with you. Come on…

Finally, the fourth sub-theme reflects the notion whereby social status is conveyed by sexual allure. This ageist approach, reducing the older LGBTQ individual to his/her sexual competencies, can even be seen among leaders or advocators of the LGBTQ communities. As expressed by Quincy, a gay male participant who had volunteered at an LGBTQ community center during his late 40s. Quincy conveyed a strong sense of solitude in a place which should have been safe and welcoming:

I always saw [ageism] in the small things, you know, like facial expressions, or responses I received to a “good morning” greeting. I was met with hostility, you’re not welcomed…I mean, these guys, who were active, were at the beginning of their independent lives at the age of 20, and I was already in my mid 40s or even closer to 50. My interpretation of this was, if you’re not sexually attractive anymore, then that determines your status, you’re not wanted anymore.

### Theme 3: Elastic and Challenging Relationships

The third theme that emerged from the interviews relates to the elastic and diverse LGBTQ relationships—that are not always acknowledged as such by outsiders. The theme attests to older LGBTQ adults’ possible [lack of] sources of support in advanced stages of life. As explained by Florine, a lesbian woman who was in a relationship but did not live with her partner:

It’s extremely important for me to emphasize that I’m not married, I have not been married. I have no children and did not want to have children. Stop asking me about [this]... Look, I’m not more of a feminist than I am a lesbian, ok?! For me, the two things go together. I want to express a different voice in me, leave me alone.

This was also conveyed by Len, a gay man, who had been married to a man for more than five years, yet preferred to continue living in separate homes:

I told him I didn’t think it [living together] was a good idea. And he agreed that our relationship was good and successful because we don’t live together. That way, neither of us missed out on anything.

The elasticity and fragility of these relationships are reflected in Raphael’s story, a gay man whose relationship had ended abruptly as a result of his plans to become a father through surrogacy.

The night before I was supposed to find out if the surrogate was pregnant or not, we went to bed, and I could see that my partner wasn’t functioning. I asked him what had happened, and he said [I’m angry about] the surrogacy. So I told him that if this is true, he can’t stay with me another minute longer. I said, You have to leave [our] home immediately. I spent half the night outside on a bench…The next morning, while I was crying, I helped him pack his belongings and leave our house.

Lack of acknowledgment of LGBTQ relationships, accompanied by a sense of isolation within an intimate relationship was articulated by Benny, a gay man who had married his partner of 10 years two years before the interview: “Since we got married, my partner’s mom doesn’t talk to me…She doesn’t speak to him either. It’s very difficult.” Similar frustration was expressed by Raphael, a gay man, when recalling the difficult conversation that he had had with his parents about him having a male partner:

Saturday morning, I called my parents and said that I needed to speak to them alone. I went over there and said, Listen, it can’t be that I live with a partner but you don’t know him. It can’t be that I can’t bring him home [to meet you]. I can’t live this way anymore.

### Theme 4: End of Life as Reverting to the Closet and to Heteronormativity

In the fourth theme in this study, the participants expressed concern relating to their aging and their becoming more dependent on others—that could force them to hide their sexual identity, to “go back into the closet,” thereby subjecting them to a heteronormative approach. They worried about this occurring due to hospitalization, or during the dying process itself. Yet most of all, this concern was expressed by the participants in relation to moving into a nursing home. 70-year-old Gadi, a gay man living in the community, said that he knew of some older gay men who lived in a nursing home, but did not reveal their sexual identity. These men felt on the outside when the other residents talked about their grandchildren. Zelda, a 75-year-old queer woman, spoke along these same lines, saying that she preferred to move into a nursing home with other lesbian friends. Otherwise, she was worried about having to once again encounter sentences such as, “Oh, you’re a lesbian. Oh this, and Oh that… All that bullshit. That’s my generation.”

Wesley, an 83-year-old gay man, explained that he does not want to go into a special nursing home for gay people: “As a gay man, I don’t see myself as being a special case who needs special care, as if I have special demands and requirements compared to other people. I think I can manage pretty well in a ‘regular’ home.” Yet on the other hand, Wesley also expressed his reverting to the closet in certain unpleasant situations, saying: “I can…pretend I’m straight…I try to think ahead, so I’ve already developed ways to protect myself from certain things.”

An additional aspect of this theme relates to near-death or death experiences. Older LGBTQ members, especially trans, expressed their fear of dying “in the wrong body” or being buried under the “wrong gender,” as a result of certain religious norms that dominate burial practices. For example, and as expressed by both Orit, 62-year-old and Tzila, 73-year-old two transwomen:

Living in a body that is right for me, that suits me, has opened up very, very significant worlds for me. Very, very deep ones…I cannot even think of going back to the state I was in before. That’s a horrific thought for me (Orit).

I saw trans people whose names were female, like Liora…But how were they buried? As male, obviously. [The cemetery workers] said, “The funeral of this man will take place shortly.” They were also covered with a *tallit*^1^ [Jewish prayer shawl]. This man, this woman, who spend her entire life fighting to be a woman. Why do you bury her as a male? You should be ashamed of yourself(Tzila).

### Theme 5: Death as a Source of Generativity and Creativity

In the fifth and final theme identified in this study, some participants expressed a more complex notion, whereby when a person nears death, they have an urge of creativity to leave behind a reflection of themselves. This may be even more pronounced for those without biological children, as seen in the small exploratory sample in this study. Moreover, this reflection could take the form of an artistic, political, or educational heritage. This notion was expressed by Benny, a gay man who was currently completing his graduate studies and had begun writing an autobiography:

Throughout my journey, I realized that although I have [my partner] X and [my non-biological child] Y, I don’t have a biological child. [After my death] I want to leave something behind…something that someone, in a few years’ time, will look on Google Scholar and find that I had written some articles on the subject.

Similar thoughts were expressed by Jack, a bisexual man who did not have a good relationship with his biological child. In addition to being involved in the LGBTQ community, he attends lectures on various areas of interests and serves as curator in contemporary art exhibitions, where he is able to express his creativity, especially as he ages. Moreover, Raphael, a gay man who is a parent via surrogacy, expressed similar concerns:

There are so many books I’d like to write, so many materials that I haven’t typed yet. My kids are my main priority, but I’m also concerned about the fate of my work. What will happen to all my archives and writings? And, you know, what will I succeed in doing, and what won’t I?

An additional source of creativity is driven by a political need to convey the history of the LGBTQ communities. This can be seen as another driving source in Benny’s desire to write an autobiography: “[In writing my autobiography], I find that the younger generations of the LGBT community don’t know what happened here in the 1980s and 1990s. They don’t know me, and very little has been written about what happened here.” Tzila, a trans woman with no children of her own, also discussed her decision to write a play about the trans community, following the suicide of a friend:

I said to myself that for her sake, I will share the story of who I am, I will provide my community with historical information about trans people. So, I wrote my life story, and if somebody wants the story, they can have it. It will be written as a theatre play. Because this kind of thing needs to be tangible and concrete.

## Discussion

This study provides an in-depth understanding of the experiences, relationships, needs and challenges of older LGBTQ members, and various meanings attached to them as they age and near death. By focusing on individual choices, obstacles, and uncertainties, the study fills a gap in the literature, thereby exposing the tensions of members of these communities among and between such communities. The aim of this study was to examine end-of-life needs, care, and planning among members of LGBTQ communities in Israel. Similar to other studies, the findings of this study convey a strong sense of loneliness as a central motif among older LGBTQ individuals, who may experience greater social discrimination than their same-age heterosexual peers, due to their sexual orientation and lifetime exposure to discrimination and stigma (Goldhammer et al., 2019; Hughes et al., 2021; Patterson et al., 2020; Tinney et al., 2015). Their loneliness is intensified by the intersectionality of older LGBTQ adults (Fredriksen-Goldsen et al., 2023), and self-alienation within and between sub-groups of their relevant communities—especially trans individuals and bisexual (e.g., between younger and older) men. This significant loneliness among the research participants harms both their diminishing wellbeing and their aging-related experiences. This is especially true given the imperfect, partial, and elastic relationships seen in this study—compared to the positive impact that valued relationships have on successful aging (Lee et al., 2017), even among adults from SGMs (Jabson Tree et al., 2021). This study significantly highlights the complexity of intimate relationships among older members of the LGBTQ communities, as reflected in their need for a certain degree of freedom from commitment (such as the decision not to have children or not to live with their partner), combined with a lack of acknowledgment of such a commitment by the close outside environment (such as their own parents).

Like previous studies conducted in Israel (Meri-Esh & Doron, 2009; Misgav, 2016a) and other countries (Lyons et al., 2022; Wight et al., 2015; Woody, 2017), the findings of this study indicate significant ageism toward older LGBTQ individuals, exhibited even by community leaders. Mostly disturbing is the ageist approach within the LGBTQ communities, especially the male-gay community, that was seen in this study; this is especially troubling given the positive and resilient impact that a connection to the gay community has on older members of that community (Brennan-Ing et al., 2022). As also reflected in our study, older members of this community are subjected to stigmas, enacted, experienced, and internalized even by activists in such communities (Herek et al., 2009). According to the minority stress model, accumulated stress (direct or indirect) that stems from prejudice, rejection, and exclusion of SGMs in a heteronormative context significantly harms subjects’ well-being (Flatt et al., 2022; Meyer, 2003).

Yet the participants conveyed that they had grown from their traumas, with their feelings of liberation assisting them in this process. They had achieved such growth by constantly promoting actions that entail choice and control throughout the course of their lives—which could be a reflection of their belonging to the silenced generation and to the pride generation (Fredriksen-Goldsen et al., 2019, 2023). Studies highlight the efficacy of developing and implementing resilience and connections to others, as a means for establishing collective purpose following trauma and difficulties (McCleary & Figley, 2017). In the current study, participants similarly spoke of how they facilitated resilience, agency, purpose finding, and even marginality, thereby creating and acknowledging meaning and enjoyment that stem from small, even day-to-day events (Meyer et al., 2011; Porter et al., 2019; Unger, 2000). As recent studies show, this may lead to a revised and more specific meaning of successful aging (Jabson Tree et al., 2021).

Moreover, the space in which older LGBTQ individuals liberate themselves allows them to move between the politics of shame to the politics of pride, as reflected through their right to not be discriminated against within their own community, and their desire to remain a part of this community (Misgav, 2016b). Especially regarding older LGBTQ individuals today, this reflects their internal conflict with social pressure to conform to normative gender expectations, while pursuing gender transition in later stages of life (Fabbre, 2017).

The findings of this study demonstrate similarities to studies conducted in other countries. For example, similar to older members of LGBTQ communities outside Israel (e.g., McDermott et al., 2018), the participants in this study conveyed their negotiating of social norms, striving to normalize processes relating to their identities, behaviors, and relationships. Yet the specific Jewish and Israeli experience was also reflected in this study—as the participants spoke of their exploring attitudes toward sexuality in older ages, procreation and elasticity of social LGBTQ relationships, and their fear of not being buried in line with their sexual orientation. The findings show how the sexual concerns of older members of the LGBTQ communities in Israel are treated from a biomedical perspective (with little relevance to quality of life and well-being), and involve stereotypes that reflect negative attitudes toward later life sexuality (Gewirtz-Meydan et al., 2020). In addition, older LGBTQ members struggle to accept or reject both pro-natalism and sexual identity, thereby re-constructing their relationships with partners as elastic and fragile—as a means for allowing such tension (Carmeli, 2020). Finally, acknowledging the Jewish Orthodox monopoly and politization of the burial process in Israel (Ben-Porat, 2013), members of the LGBTQs communities, especially trans people, fear having to “go back into the closet” and being buried in contradiction to their personal identity. Theoretically, these findings could support the social identity theory (Tajfel & Turner, 1986), whereby identity is a process in which the self struggles to connect between forms of sociopsychological thoughts and explanations and the complexities of social life (Duveen, 2013).

Similar to previous findings (Löf & Olaison, 2020; Mahieu et al., 2019), the participants fear being discriminated against, forced even to revert to the closet—rendering them spending their later years living in a heteronormative environment, dependent on social services and others. The aging of LGBTQ members demonstrates the fluidity and instability of the “closet” and gender identities and sexuality throughout their lifetime (Butler, 2011). Such experiences challenge heteronormative manipulations that strive to eliminate such confusing circumstances. The more optimistic and creative places in which LGBTQ member find meaning in later life indicate the need for queer theories that combine studies on age and aging (Hurd et al., 2022) and the more general connection between trauma and vulnerability—and between liberty and resilience. In line with the concept of queering aging futures (Sandberg & Marshall, 2017), this study shows that the creativity of older adults in LGBTQ communities enables its members to reconstruct and rethink aging and death. This goes beyond the promised hetero-happiness that underpins the mainstream concept of successful aging. It allows the reimagining and retelling of past-future stories, and of the history of LGBTQ communities, that are often conveyed through autobiographies and plays (Jones et al., 2023). At the theoretical level, the current study refines different understandings of life courses, demonstrating how living could be desirable even in old age; the study thereby rejects the binary concepts that shape successful vs. failed aging and dying. As such, ageism and end-of-life are characterized not only by finality and extinction, but also by hope and revival.

### Limitations

This article contributes to the literature on aging among members of LGBTQ communities in Israel. Yet a number of research limitations should be addressed. First, convenience and snowball sampling methods were employed for recruiting participants. While these methods may lead to certain bias for recruiting participants that are “similar” or homogenous, they may be highly valuable in providing access to participants when researching difficult or sensitive issues—such as aging and sexual and gender minorities (Parker et al., 2020). Additionally, to increase the participant diversity, requests to participate were also employed.

### Conclusions and Implications

In summary, this study demonstrates that loneliness is an existential experience, one that is exacerbated by the intersectionality of members of LGBTQ communities in Israel. Moreover, the chosen family members play a minimal role in the end-of-life care of their loved ones. The participants expressed a certain degree of ambivalence toward welfare, social, and housing services for the aging, yet explicitly spoke of their fear of being discriminated against, rendering them having to re-enter the closet as they age. The findings of this study could therefore be useful in promoting actions for mitigating homophobic and transphobic barriers against older GSM members, while enhancing LGBTQ-sensitive social, healthcare, and housing services for older adults who are members of these communities. Examples of such affirming solutions can be seen in the USA, in the Crotona Pride House in the Bronx and in the Stonewall House in Brooklyn (SAGEServe, 2024). Yet additional research is needed to explore the experiences and perceptions of consumers of such services.

Another important implication of this study relates to the finding whereby ageism and end-of-life do not only represent finality and extinction, but also hope and revival. Those who work with older members of the LGBTQ communities should seek additional ways for enhancing the agency and creativity of these individuals, to significantly contribute to their daily experiences—even in difficult situations. Moreover, the unique challenges that are associated with the familial and social support of older adults from LGBTQ communities, and their implications on care, deserve a more central place in caring for these individuals and in securing sufficient resources. Doing so could enhance their self-management and resilience, as a means for overcoming obstacles related to aging and end of life. Finally, further research is needed on the varied sources of support for older members of LGBTQ communities, as their findings could be applied in both clinical practices and in intervention programs.

## Data Availability

The data and research material are confidential and with author.

## Appendix: The Interview Guide

### Part I: Biographic Questions

Please refer to your age, sexual orientation, gender identity, marital status, parental status, education level, work experience, health status, nationality, geographic region of residence, and any other demographics that you would like to share with me.

### Part II: Sexual Orientation

- How do you define your sexual orientation?
- When did you know that you were L/G/B/T/Q?
- Please tell me about your "coming out" process.
- What is it like for you, living as an L/G/B/T/Q/ man/woman?
- To what extent do you accept yourself as an L/G/B/T/Q?

### Part III: Perceptions of Ageing and End-of-Life

- What does end-of-life mean to you?
- In your view, what characterizes this stage?
- What thoughts do you have regarding this stage?
- Have you made or considered making any plans for this stage?
- Do you think that preparing for end-of-life is different for people who are not heterosexual? If so, how?
- To what extent do you fear getting to this stage?
- What are you most afraid of?
- What do you expect will happen to you at that stage?
- What would you like to happen to you at that stage?
- Who would you like to be with at end-of-life?
- How meaningful is that for you?

### Part IV: Experiences with the Health Sector

- How would you describe your experiences with healthcare providers?
- To what extent has your sexual orientation influenced your interactions with healthcare providers? If it has, please provide an example.
- How, if at all, will your sexual orientation affect the care you will receive in end-of-life?”

### Part V: Social and Organizational Surroundings

- Who do you talk to about end-of-life issues?
- Under what circumstances?
- Do you think that you would like to share your thoughts about these issues with other people? If so, who would you like to confide in?
- How would you like to acquire knowledge that you are currently lacking about ageing and end-of-life?

### Part VI

- Is there anything that you would like to address here that has not been raised in the interview?
- Did you have any thoughts during the interview that you would like to share with me?
- Are there any questions you would like to ask me, related to the research topic?

Thank you so much for your participation.

## Footnotes

1 According to Jewish custom, deceased men are buried in a *tallit*, a prayer shawl for men.
